# Risk Factors Associated with the Occurrence of Gastrointestinal Helminths among Indigenous Donkeys (*Equus asinus*) in Northeastern Nigeria

**DOI:** 10.1155/2016/3735210

**Published:** 2016-06-06

**Authors:** Saleh Mohammed Jajere, Jallailudeen Rabana Lawal, Amina Mohammed Bello, Yakaka Wakil, Usman Aliyu Turaki, Ibrahim Waziri

**Affiliations:** ^1^Department of Veterinary Public Health and Preventive Medicine, Faculty of Veterinary Medicine, University of Maiduguri, PMB 1069, Maiduguri, Borno State, Nigeria; ^2^Department of Veterinary Medicine, Faculty of Veterinary Medicine, University of Maiduguri, PMB 1069, Maiduguri, Borno State, Nigeria; ^3^Department of Animal Science, Faculty of Agriculture, Federal University Kashere, PMB 0182, Kashere, Gombe State, Nigeria; ^4^Ministry of Animal Husbandry and Nomadic Affairs, GSADP Block, Off Bauchi Road, PMB 0010, Gombe, Gombe State, Nigeria

## Abstract

*Aim*. This survey study was conducted from April 2014 through March 2015 in Bauchi, Yobe, and Gombe states, northeastern Nigeria, to explore the risk factors associated with the occurrence of gastrointestinal helminthosis among indigenous donkeys (*Equus asinus*).* Materials and Methods*. A total of six hundred fresh faecal samples were randomly collected from indigenous donkeys of varying age, sex, and settlements. Simple flotation and sedimentation techniques were used for the detection of helminths eggs.* Results*. Three gastrointestinal nematode parasites were encountered including* Strongyle*,* Parascaris equorum*, and* Oxyuris equi*. An overall prevalence of 98.3% was obtained, of which 78.3%, 40.3%, and 17.5% were, respectively, from* Strongyle*,* Parascaris equorum*, and* Oxyuris equi*. Age, sex, and season were not statistically associated with the risk of helminth infection as were the different study areas (*p* > 0.05). However, body condition score, settlement, anthelminthic medication history, and management practices were significantly associated with the risk of gastrointestinal helminthosis. Statistically high prevalence of helminthic infections was observed in donkeys, with poor (thin) body condition, from rural settlements, that were not dewormed and raised under poor management systems (*p* < 0.001).* Conclusion*. It is concluded from the study that gastrointestinal helminths particularly* Strongyle* were endemic among the indigenous donkeys in northeastern Nigeria. Further control and preventive measures were discussed.

## 1. Introduction

Donkeys (*Equus asinus*) are among the early-domesticated equines that have been a beast of burden for thousands of years [1]. Currently, there are about 44 million donkeys distributed throughout the world [2]. In Africa, the donkey population is estimated at 13 million [3]. Despite the increase in mechanization throughout the world, donkeys still have a prominent position in the agricultural systems of many developing countries [4–6]. Donkeys represent an important aspect of small holder farming system especially in rural communities and hamlets, where there exist poor road networks or even nonexisting roads; they are used for conveying people, goods, and farm inputs and outputs to and from farms [7, 8]. Unfortunately, Donkeys are left malnourished and underfed, subjected to different conditions of hardships like maltreatment, poor management conditions, and overladen [9].

Donkey still remains one of the most underappreciated animals and an important draught one, serving a key role in the agricultural economy of the developing world including Africa [10, 11]. They are often engaged in work for long hours and when set free, they are left to browse and feed on less nutritive garbage. This has been linked with potential negative effect especially on their welfare and health status [12, 13].

Donkeys like other domesticated animals are vulnerable to parasitic, bacterial, fungal, and viral diseases [14, 15]. Among parasitic diseases are the haemoparasites such as babesiosis, trypanosomiasis, and gastrointestinal helminthosis, which are considered silent killers of the animal [16–19]. Donkeys are hosts to a variety of gastrointestinal tract parasites of the family Strongylidae, commonly called* Strongyle* nematodes or* Strongyles* [20]. The most common gastrointestinal parasites of donkeys include large and small* Strongyles*,* Ascaris*, pinworms (*Oxyuris equi*),* Gasterophilus*, lung worms, and fluke and tape worms which are by far the commonly encountered parasites in most veterinary parasitological examinations of donkeys [6, 14, 21, 22]. Some of these gastrointestinal tract parasites, especially* Strongyles*, are active bloodsuckers; where there is heavy parasitic burden, the commonly encountered manifestations are anaemia, weakness, emaciation, and sometimes colic and diarrhea [23]. The intestinal mucosa is damaged where the worms attach themselves and suck blood. These parasites usually deprive their host animals of sufficient and adequate absorption of digestive nutrients, which in the long run lead to retarded growth and impaired productivity, reduced work output, discomfort, and pains and sometimes death ensued [24, 25]. The various degrees of damage caused by the gastrointestinal parasites of donkeys depend on factors such as the species affected and nutritional and immune status of the animal [25].

Although many studies were conducted on the prevalence and risk factors associated with gastrointestinal parasitism in horses in northeastern Nigeria, there are few researches on the risk factors, epidemiology, and prevalence of gastrointestinal parasites among indigenous donkeys (*Equus asinus*) in the region. Moreover, the huge numbers and increasing importance of donkeys in the economy of the semiarid zone of Nigeria particularly seen in hamlets and rural communities where these animals are used for variety of purposes including transporting people and farming inputs and outputs, make them important in these communities. Therefore, this study was designed to determine the prevalence and associated risk factors of gastrointestinal helminths among working donkeys in northeastern Nigeria. It is expected to provide baseline epidemiological data on prevalence and risk factors for which further research would be conducted in elucidating gastrointestinal helminthoses among indigenous donkeys in the region.

## 2. Materials and Methods

### 2.1. Study Areas

This study was conducted in some parts of three provinces of the northeastern Nigeria comprising Bauchi, Yobe, and Gombe states. Gombe state, a multiethnic city, is located in the northeastern zone of Nigeria, with Gombe city as its capital. It is located at latitudes 9°30′ and 12°30′N and longitudes 8°45′ and 11°45′E. It shares common borders with Borno, Bauchi, Yobe, Taraba, and Adamawa states due to its location within the expansive savannah zone. The state has an area of 20,265 km^2^ and an estimated population of 2,353,000 people as of 2006. Gombe state has two distinct climates, rainy season spanning from April to October and dry season spanning from November to March with an average rainfall of 850 mm (https://en.wikipedia.org/wiki/Gombe_State).

Bauchi state is located in the northeastern zone of Nigeria at latitudes 9°3′ and 12°3′N and longitudes 8°50′ and 11°E. It has a total landmass of 49,119 km^2^ and an estimated human population of 4,653,066 as of 2006 census. The state shares border with seven states, namely, Taraba and Plateau state to the south, Yobe and Gombe state to the east, Kano and Jigawa state to the north, and Kaduna state to the west. The state has two distinctive kinds of vegetation: the Sudan savannah covering the entire southern part and Sahel savannah from the middle of the state as you go from the southern to the northern part of the state. Majority of the northern part is sandy, while the southern part is mountainous resulting from continuation of the Jos plateau. The rainfall ranges from 700 mm in the northern part to 1300 mm per annum seen in the southern part of the state. Rain starts early from April in the southern part, which is heavier and lasts longer. However, in contrast, rain starts lately around June or July and lasts shorter (https://en.wikipedia.org/wiki/Bauchi_State).

Yobe state is located in the arid zone of northeastern Nigeria within latitude 11°5′ north and longitude 13°10′ east. It has an estimated human population of 2,757,000 as of 2011 and covers a land mass area of 45,502 km^2^. Damaturu is its capital city. The state shares an international border with Diffa and Zinder regions of the Niger Republic to the north. In addition, it shares border with Nigerian states of Bauchi, Borno, Gombe, and Jigawa. It lies within the dry savanna belt. The climate is mostly dry and hot throughout the year with milder climate seen in the southern part of the state. The short rainy season starts lately around June and ends in September.

### 2.2. Study Design, Baseline Characteristics, and Sample Collection

Cross-sectional study was conducted on six hundred randomly selected indigenous donkeys from Bauchi (*n* = 200), Gombe (*n* = 200), and Yobe states (*n* = 200) in northeastern Nigeria. The sample comprises 364 males and 236 females: 103 young, 416 adult, and 81 older Donkeys. Faecal samples were collected, while data on sex, age, body condition scoring, season of the year, and so forth were also recorded accordingly. Random faecal samples were collected directly from the rectum of the donkeys after a well physical restrained technique by owners and voluntary veterinary attendants (especially the temperamental animals) using sterilized disposable hand gloves. About 10 grams of fresh faecal samples was collected. Some faecal samples were also collected from donkeys with previous history of medications. Each sample was labeled with necessary information and then kept in icebox and immediately transported to parasitology diagnostic laboratory for examination. Other relevant information on management systems was also noted at the time of sampling.

### 2.3. Age and Body Condition Estimation

The age was determined using birth records gathered from information obtained from the owners' and dentition characteristics [26] and this was categorized as young (<12 months) (*n* = 103), adult (>1 year) (*n* = 416), and old (>6 years) (*n* = 81). These age classifications were described elsewhere [27–29]. The body condition scoring was based on the criteria of NEWC [30] and body condition of animals was classified into poor, medium (moderate), and good.

### 2.4. Faecal Analysis and Coprology

Samples were kept in refrigerator at 4°C when immediate processing was not possible but processed within 48 hrs. Some samples were held using 10% formalin solution. Parasitological examination was carried out by direct smear, sedimentation, and flotation techniques, following the standard procedures for the screening of parasites and microscopic examination (10x and 40x objectives) as described by Soulsby [31]. Identification of the helminth parasite eggs in the faeces of the donkeys was evaluated using the coprological flotation and sedimentation techniques [32]. The flotation fluid used in the present study was supersaturated solution of sodium chloride (NaCl) salt prepared in the laboratory.

### 2.5. Data Analysis

The raw data were cross-tabulated initially using Microsoft Office Excel version 2011 to obtain proportions and prevalence of infection. This was later imported into SPSS statistical software version 22 for chi-square analysis and Fisher's exact test in order to determine the strength of association between the dependent and independent variables. The association is considered significant at *p* < 0.05.

## 3. Results and Discussion

An overall prevalence of 98.3% was obtained (Table 1), of which 78.3%, 40.3%, and 17.5% were, respectively, from* Strongyle* spp.,* Parascaris equorum*, and* Oxyuris equi* (Table 2). Based on the study areas, prevalence of gastrointestinal helminthosis ranged from 99.0% observed in both Yobe and Gombe states to 97% in Bauchi state. Infection rates were not statistically significant across the study areas (*p* > 0.05). The infection burden with the nematodes was manifested in both single and mixed infections (Table 3).

The relative frequency of single infection ranged from 47.8% seen in* Strongyle* to 5.4% as seen in* Oxyuris equi*, while it ranges from 19.5% in* Strongyle* +* Parascaris equorum* to 5.8% seen in* Strongyle* +* Oxyuris equi* in the mixed infection (Table 3). The age-, season-, sex-specific prevalence rates were similar across each of these categories and were not statistically significant (Table 1). However, donkeys sampled from rural areas, not previously dewormed, with thin body condition score and raised under poor management systems had high infection rates and were significantly associated with the risks of gastrointestinal helminthosis (*p* < 0.001) (Table 1).  Table 4 presents the distribution of the gastrointestinal nematodes of indigenous donkeys with the occurrence of* Strongyle* eggs significantly higher compared with the other nematodes encountered.

Parasitic helminths continue to be the major constraints affecting the health and working performance of donkeys worldwide. They cause various degrees of damage depending on the species and nutritional and the immune status of the donkey. They decrease the performance and productivity through reduction of body weight or retarded weight gain and even death in acute cases. The overall prevalence of helminth parasites among donkeys reported is 98.3% (Table 1), which agrees with reports by Ayele et al. [28], Ibrahim et al. [14], and Wannas et al. [33] who reported respective prevalence of 98.2%, 96.9%, and 100% in Dugda Bora District, southern Ethiopia, and Al Diwaniyah Governorate, respectively. However, it is relatively lower than 90%, 86.5%, 75.9%, and 55.7% prevalence rates as reported by Samuel et al. [6], Parsani et al. [9], Getachew et al. [34], and Muhammad et al. [35] in Kombolcha Town, North Gujarat, Ethiopia, and Pakistan, respectively. These differences might be connected with factors such as variations in sample sizes, sampling periods, deworming strategy, accessibility to veterinary clinics, and methods employed for sample analysis, which may affect the prevalence rate. This may also be partly explained by the nutritional status of donkeys in the respective study areas, management systems, and environmental factors that can influence the level of immunity to infection and manifestation of the parasitic infections. In the present study,* Strongyle*,* Parascaris equorum*, and* Oxyuris equi* were reported with respective prevalence of 78.3%, 40.3%, and 17.5% (Table 2). Several studies highlighted the significance of these nematodes as important gastrointestinal parasites of donkeys [6, 13, 14, 20, 36, 37].

Considering a single gastrointestinal nematode infection,* Strongyle* spp. egg was prevalent and dominant and the most encountered nematode species examined from all samples collected from the study areas (Table 3). This agrees with previous studies by Saeed et al. [38], Wannas et al. [33], Mezgebu et al. [25], and Tesfu et al. [37] which further explained the predomination of* Strongyle* egg with prevalence rates of 58.5%, 57.14%, and 66.67%, respectively, over* Parascaris* spp. eggs. In the current study,* Oxyuris equi* with prevalence rate of 5.5% was in accord with the study of Ayele et al. [28] and Umur and Açici [24], where both reported rates of 6% and 6.45%, respectively. However, Yoseph et al. [29] and Ibrahim et al. [14] reported respective rates of 32.4% and 31.8% of* Oxyuris equi* in their study. This could probably be due to influence of climatic conditions on the dynamics of egg expulsion [39]. The relative frequency for the occurrence of mixed infection in the present study agrees with similar study by Samuel et al. [6].

Age-specific prevalence rates revealed a slightly higher rate among young donkeys (Table 1). This agrees with the study by Regassa and Yimer [36] and Tesfu et al. [37], which reported high prevalence of gastrointestinal nematodes in younger donkeys as compared to the adult age groups. This might be associated with apparent inability of the younger age groups to develop adequate acquired immunity predisposing them to high risks of severe infection with gastrointestinal nematode parasite when compared with adult donkeys. Higher infection rates and more severe infections reflect lack of immunity in younger population [40].

Sex was not significantly associated with infection rates, as both sexes share equal chances of acquiring nematode infection, because they are reared and grazed on the same pasture without sex discrimination. It could also result from decreased infection resistance at the time of parturition and during early lactation. The periparturient relaxation of resistance to gastrointestinal nematode infection has been reported in the female donkeys [40].

Body condition score was significantly associated with infection rates (Table 1). Donkeys with poor body condition score were significantly associated with high parasitic activities in their gastrointestinal tracts depriving them of adequate absorption and assimilation of digested nutrients resulting in emaciation and cachexia. Similarly, Ayele et al. [28], Ibrahim et al. [14], Worku and Afera [21], Tesfu et al. [37], and Samuel et al. [6] all reported that the body condition score is a reliable indicator of parasitic burden and can serve as a useful indicator used to identify donkeys that require an immediate attention for anthelminthic remedies.

Mean egg counts were high among donkeys from rural areas compared with those from urban settlements (Table 1). Donkeys in the rural areas are subjected to prolonged working time, exposed to harsh environmental temperature, and overloaded without adequate observation of their welfare. Donkeys in rural areas are generally malnourished and underfed, left to graze after hard labour on dry poorly nutritive pastures especially during the dry season, without any supplementation with essential nutrients that will improve their health status. Consequently, they become immunosuppressive and therefore vulnerable to infection with helminthic parasites. In addition, most of these rural areas lack veterinary facilities or clinics for routine medical attention to these donkeys [11, 13, 25].

Donkeys with no history of recent deworming had significantly high mean egg counts compared with those routinely dewormed. This implies that routine deworming of donkeys with effective anthelminthic will reduce egg burden, thereby safe-guarding the animals from nematode infections. Muhammad et al. [35] reported respective 73.21% and 96.42% mean reduction in number of eggs per gram of faeces on days 7 and 14 after medication with an efficacious anthelminthic. Binev et al. [41] and Seri et al. [42] also reported 96% and 100% elimination of nematodes egg burden in donkeys after day 14 after treatment, respectively.

Low mean egg counts were observed in donkeys reared under good management systems provided with good feed, watering, and veterinary attention as compared with those reared under poor management conditions. Animals reared under a good management system tend to possess strong immunity and demonstrate resistance to infectious diseases [11, 22, 43].

In conclusion, gastrointestinal helminth parasites are endemic among the indigenous donkeys of northeastern Nigeria, with* Strongyle* as frequently encountered compared with the other nematodes (Tables 2 and 4). Therefore, it is recommended that donkey owners be trained on how to improve on the management systems, especially as regards provision of adequate nutritional supplements to donkeys so that they can have good body condition that will in the long run confers some level of resistance against helminths infection. Donkeys should be well fed and served with fresh clean water following hard labour. All newly introduced donkeys into the herd meant for restocking or breeding purposes must be quarantined, properly screened, and treated to prevent environmental contamination with helminth parasites. Further studies are recommended on the impact of parasitic infections on the health status, working efficiency, reproductive efficiency, draftability, and longevity of donkeys.

## Figures and Tables

**Table 1:** Risk factors associated with the occurrence of gastrointestinal helminthosis in northeastern Nigeria.

Risk factors	Number of donkeys sampled	Number of positives	Prevalence (%)	*p* value
*Age*				
Young (<12 mo)	103	103	100.0	
Adult (>1 yr)	416	407	97.8	0.292
Old (>6 yrs)	81	80	98.8	
*Sex*				
Male	364	357	98.1	0.542
Female	236	233	98.7	
*Body condition score*				
Thin	355	355	100.0	
Medium	150	150	100.0	
Fat	95	85	89.5	<0.001
*Settlement *				
Urban	120	110	91.6	<0.001
Rural	480	480	100	
*Season*				
Rainy	300	298	99.3	
Dry	300	292	97.3	0.056
*Anthelminthic medication history*				
Dewormed	31	21	67.7	<0.001
Not dewormed	569	569	100	
*Management system* ^*∗*^				
Poor	517	517	100	
Moderate	52	52	100	
Good	31	21	67.7	<0.001
*Overall*	*600*	* 590 *	* 98.3*	

^*∗*^Management system (feeding, shelter, healthcare, welfare, etc.).

**Table 2:** Prevalence of gastrointestinal nematode parasite of donkeys (*Equus asinus*) in northeastern Nigeria (*n* = 600).

Parasites encountered	Number of positives	Prevalence [% (95% CI)]
*Strongyle*	470	78.3 (75.00–81.60)^*∗*^
*Parascaris equorum*	242	40.3 (36.38–44.22)
*Oxyuris equi*	150	17.5 (14.46–20.54)

^*∗*^Statistically significant at *p* < 0.05.

**Table 3:** The relative proportion of gastrointestinal nematode parasites of donkeys (*Equus asinus*) in northeastern Nigeria.

Nematodes	Number of positives	Relative frequency (%)
*Strongyle*	282	47.8
*Parascaris equorum*	88	14.9
*Oxyuris equi*	32	5.4
*Strongyle *+ *Parascaris equorum*	115	19.5
*Strongyle *+ *Oxyuris equi*	34	5.8
*Strongyle* + *Parascaris equorum *+ *Oxyuris equi*	39	6.6
*Overall*	*590*	*100*

**Table 4:** Burden of helminth infection according to the risk factors among donkeys (*Equus asinus*) in northeastern Nigeria.

Risk factors (*n*)	% distribution of helminths based on species					
	*Strongyle* ^a^	*Parascaris equorum*	*Oxyuris equi*	*Strongyle + P. equorum*	*Strongyle + O. equi*	*Strongyle + P. equorum + O. equi*
*Age*						
Young (<12 mo) (103)	47 (45.6)	14 (13.6)	8 (7.8)	22 (21.4)	4 (3.9)	8 (7.8)
Adult (>1 yr) (407)	197 (47.4)	62 (14.9)	17 (4.1)	80 (19.2)	26 (6.3)	25 (6.0)
Old (>6 yrs) (80)	38 (46.9)	12 (14.8)	7 (8.6)	13 (16.0)	4 (4.9)	6 (7.4)
*Sex*						
Male (357)	172 (47.3)	56 (15.4)	16 (4.4)	68 (18.7)	21 (5.8)	24 (6.6)
Female (233)	110 (46.6)	32 (13.6)	16 (6.8)	47 (9.9)	13 (5.5)	15 (6.4)
*BCS* ^b^						
Thin (355)	161 (45.4)	59 (16.6)	17 (4.8)	75 (21.1)	20 (5.6)	23 (6.5)
Medium (150)	78 (52.0)	19 (12.7)	9 (6.0)	25 (16.7)	9 (6.0)	10 (6.7)
Fat (85)	43 (45.3)	10 (10.5)	6 (6.3)	15 (15.8)	5 (5.3)	6 (6.3)
*Settlement*						
Urban (110)	52 (43.3)	14 (11.7)	8 (6.7)	23 (19.2)	6 (5.0)	7 (5.8)
Rural (480)	230 (47.9)	74 (15.4)	24 (5.0)	92 (19.2)	28 (5.8)	32 (6.7)
*Season*						
Rainy (298)	147 (49.0)	41 (13.7)	14 (4.7)	50 (16.7)	18 (6.0)	28 (9.3)
Dry (292)	135 (45.0)	47 (15.7)	18 (6.0)	65 (21.7)	16 (5.3)	11 (3.7)
*Anthelminthic history*						
Dewormed (21)	12 (38.7)	4 (12.9)	0 (0.0)	3 (9.7)	2 (6.5)	0 (0.0)
Not dewormed (569)	270 (47.5)	84 (14.8)	32 (5.6)	112 (19.7)	32 (5.6)	39 (6.9)
*Management system* ^b^						
Poor (517)	245 (47.4)	73 (14.1)	25 (4.8)	107 (20.7)	31 (6.0)	36 (7.0)
Moderate (52)	25 (48.1)	11 (21.2)	7 (13.5)	5 (9.6)	1 (1.9)	3 (5.8)
Good (21)	12 (38.7)	4 (12.9)	0 (0.0)	3 (9.7)	2 (6.5)	0 (0.0)

^a^Statistically significant at *p* < 0.05.

^b^BCS: body condition score.
